# Ethyl Carbamate in Alcoholic Beverages from Mexico (Tequila, Mezcal, Bacanora, Sotol) and Guatemala (Cuxa): Market Survey and Risk Assessment

**DOI:** 10.3390/ijerph6010349

**Published:** 2009-01-20

**Authors:** Dirk W. Lachenmeier, Fotis Kanteres, Thomas Kuballa, Mercedes G. López, Jürgen Rehm

**Affiliations:** 1 Chemisches und Veterinäruntersuchungsamt (CVUA) Karlsruhe, Weissenburger Strasse 3, 76187 Karlsruhe, Germany; E-Mail: thomas.kuballa@cvuaka.bwl.de (T. K.); 2 Centre for Addiction and Mental Health (CAMH), 33 Russell Street, Toronto, ON, M5S 2S1, Canada; E-Mail: fotis@fotiskanteres.com (F. K.); 3 Unidad de Biotecnología e Ingeniería Genética de Plantas, Centro de Investigación y Estudios Avanzados del IPN, 36500 Irapuato, Gto., Mexico; E-Mail: mlopez@ira.cinvestav.mx (M. G. L.); 4 Dalla Lana School of Public Health, University of Toronto, 55 College Street, Toronto, ON, M5T3M7, Canada; E-Mail: jtrehm@aol.com (J. R.); 5 Institute for Clinical Psychology and Psychotherapy, TU Dresden, Chemnitzer Strasse 46, 01187 Dresden, Germany

**Keywords:** Ethyl carbamate, urethane, alcoholic beverages, risk assessment, Mexico, Guatemala, carcinogens, gas chromatography, mass spectrometry, food contamination, food analysis, sugarcane, agave, tequila, mezcal, cuxa, unrecorded alcohol

## Abstract

Ethyl carbamate (EC) is a recognized genotoxic carcinogen, with widespread occurrence in fermented foods and beverages. No data on its occurrence in alcoholic beverages from Mexico or Central America is available. Samples of agave spirits including tequila, mezcal, bacanora and sotol (n=110), and of the sugarcane spirit cuxa (n=16) were purchased in Mexico and Guatemala, respectively, and analyzed for EC. The incidence of EC contamination was higher in Mexico than in Guatemala, however, concentrations were below international guideline levels (<0.15 mg/L). Risk assessment found the Margin of Exposure (MOE) in line with that of European spirits. It is therefore unlikely that EC plays a role in high rates of liver cirrhosis reported in Mexico.

## 1. Introduction

Ethyl carbamate (urethane, C_2_H_5_OCONH_2_, CAS # 51-79-6) is a recognized genotoxic carcinogen, of widespread occurrence in fermented foods and beverages [1–6]. Public health concerns about the presence of this substance in alcoholic beverages began in 1985, when relatively high levels were detected by Canadian authorities in imported alcohol products, including German fruit spirits [7]. Canada proceeded to establish an upper limit of 0.15 mg/L for ethyl carbamate in distilled spirits and 0.4 mg/L for ethyl carbamate in fruit spirits [8]. However, currently there are no harmonised maximum levels for ethyl carbamate provided by the Codex Alimentarius, the European Union, nor the two countries under study.

The International Agency for Research on Cancer (IARC) recently upgraded its classification of ethyl carbamate to group 2A (probably carcinogenic to humans). This reflects increasing evidence on the significant similarities between rodents and humans regarding the metabolic pathways of the activation of ethyl carbamate, whereby the formation of proximate DNA-reactive carcinogens, hypothesized to play a major role in ethyl carbamate-induced carcinogenesis in rodent cells, is also likely to occur in humans [9].

The Joint FAO/WHO Expert Committee on Food Additives (JECFA) has concluded that exposure to ethyl carbamate in food is of low concern [10]. However, the inclusion of alcoholic beverages into this model sets the estimated Margin of Exposure (MOE) to 3,800, a value of concern that necessitates mitigative measures for the reduction of ethyl carbamate concentrations in certain alcoholic beverages (for summary of mitigation measures see Ref. [9]). The European Food Safety Authority (EFSA) recently confirmed this evaluation, also noting that the MOE for high consumers of fruit spirits was less than 600, indicating an even greater public health concern [8]. The JECFA evaluation was based on data from the USA, the UK and Japan, whereas the EFSA evaluated data from Europe and North America. Evidence in the international literature (as summarized in Ref. [9]), which is generally restricted to European-style alcoholic beverages, indicates that the ethyl carbamate problem may be limited to certain fruit spirits (mainly stone-fruit spirits).

The alcohol market in Mexico ranges from international commercial alcohol including spirits and beer, to locally produced alcohol such as tequila, mezcal, and pulque (all agave based). While the Mexican Tequila Regulatory Council (TRC) monitors tequila, there are many small-scale independent and unrecorded alcohol operations producing mezcal and other local spirits (see 2.1).

There is currently no systematic data available on ethyl carbamate levels in locally produced alcoholic beverages from Mexico and Central America, including Guatemala. Our interest in ethyl carbamate in alcoholic beverages in this region comes from evidence of high rates of liver cirrhosis in Mexico [11]. This public health concern cannot be accounted for by volume of alcohol consumption, which is lower in Mexico when compared to many other countries with markedly lower liver cirrhosis rates, nor alternative explanations [11, 12]. An exception is limited evidence pointing to water contamination in locally produced alcohol pulque (fermented agave juice) [13]. Liver cirrhosis rates have been found to vary with consumption of pulque [14, 15].

Ethyl carbamate is not only carcinogenic but also a known liver toxic agent in humans [16]. Animal experiments point to complex interactions between ethanol and ethyl carbamate, e.g. in decreased first-pass hepatic clearance [17]. This study investigates ethyl carbamate in agave spirits from Mexico and sugarcane spirits from Guatemala and the possible risk to public health with special regard to liver damage.

## 2. Materials and Methods

### 2.1. Samples

**Figure 1:**
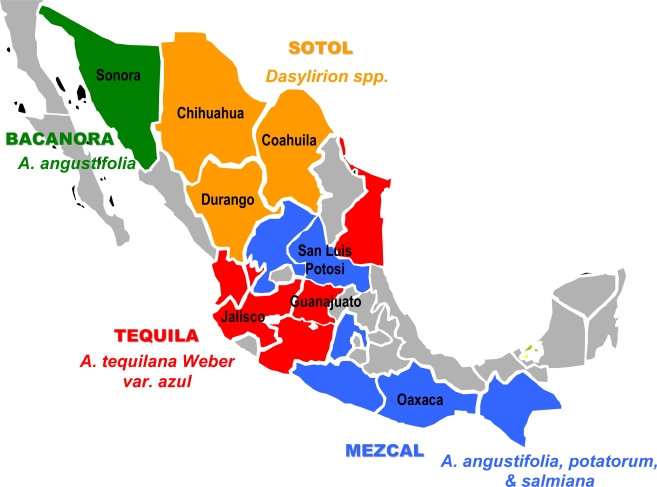
Sampling regions of Mexican agave spirits.

The Mexican alcohol samples were purchased between 2005 and 2008 from local shops, markets and distilleries. Authentic tequila samples of “100% agave” and “mixed” categories were available from controlled tequila production facilities (n=32). Tequila is the most well known agave derived mezcal (*Agave tequilana* Weber var. azul, Agavaceae)*,* and is from a strictly defined geographic region of the Mexican State of Jalisco, in West-Central Mexico. Tequilas were purchased in Jalisco and Guanajuato. Sotol came from the States of Chihuahua, Coahuila and Durango (n=16) and bacanora from Sonora (n=13). Mezcal (n=35) came from the states of Oaxaca (including samples from distilleries in the town of Santiago Matatlan, the self proclaimed ‘Mezcal capital of the world’), San Luis Potosi, and Guanajuato. The sampling regions in Mexico and the different agave species used for production of the spirits are shown in Figure 1. Imported tequila products available for regular retail in Germany were also included in the study (n=14). Previously, a sub-group of the Mexican samples was characterized in more detail in regards to volatile and anionic composition [18].

The Guatemala sampling was restricted to cuxa, a distilled sugarcane alcohol produced and distributed both commercially and clandestinely in Nahualá, an indigenous Mayan municipality located in the western highlands of Guatemala. Sampling occurred between November 2007 and April 2008 in the urban center of the municipality and nearby outlying settlements (10 km radius). Within the study area we identified thirteen cuxa distribution points, three of which were also production operations; twelve were visited for sampling. The samples included both clandestinely (n=11) and commercially (n=5) produced cuxa. Alcohol in Mayan Guatemala was previously described in greater detail [19].

### 2.2. Analysis of Ethyl Carbamate

The analysis of ethyl carbamate involved the combination of previously published procedures of the Extrelut^®^ extraction procedure of Baumann and Zimmerli [20] with the modifications of Mildau *et al*. [21], and tandem mass spectrometry (GC/MS/MS) according to Lachenmeier *et al*. [22]. For sample preparation, spirit (20 mL) was mixed with ethyl carbamate-d_5_ (1 mg/mL, 50 μL) that was synthesized according to Funch and Lisbjerg [23], and directly applied to the extraction column. The Extrelut^®^ column was wrapped in aluminium foil to eliminate the possibility of light-induced ethyl carbamate formation during extraction. After 15 min of equilibration, the column was washed with *n*-pentane (2 x 20 mL). The analytes were then extracted using dichloromethane (3 x 30 mL), and the eluates combined in a brown flask and reduced to 2–3 mL in a rotary evaporator (30°C, 300 mbar). After which, the solution was adjusted to 10 mL with ethanol in a measuring flask and directly injected into the GC/MS/MS system. The recovery of ethyl carbamate was 100.4±9.4%. The limits of detection (LOD) and quantitation (LOQ) were 0.01 and 0.04 mg/L of ethyl carbamate, respectively. The precision (expressed as coefficient of variation) and the trueness (expressed as bias) did not exceed 7.8% (intraday) and 10.1% (interday), and 11.3% (intraday) and 12.2% (interday) respectively [22].

### 2.3. Statistics

The survey data were evaluated using Origin Pro v7.5 (OriginLab Corporation, Northampton, MA, USA). Statistical significance was assumed at below the 0.05 probability level. Groups of two cases were compared using t-tests. One-way ANOVA was used to test whether three or more cases had the same mean, including the Bonferroni *post hoc* means comparison.

### 2.4. Approach for Risk Assessment

Analysis was conducted according to the European Food Safety Authority (EFSA) harmonised approach for the risk assessment of substances which are genotoxic and carcinogenic [24]. The EFSA has developed and recommends an approach known as the Margin of Exposure (MOE). This approach uses the value of doses of substances that have been observed to cause low but measurably harmful responses in animals as a reference point, and compares this with relevant substance-specific dietary intake estimates in humans, taking into account differences in consumption amounts and patterns.

To obtain the MOE, the Benchmark Dose Lower Confidence Limit (BMDL) of 10% is suggested. The BMDL value for ethyl carbamate obtained by JECFA for the incidence of alveolar and bronchiolar neoplasms (the most sensitive endpoints) in male and female mice is 0.3 mg/kg bw/day [10]. The EFSA used the same BMDL value for their risk assessment of ethyl carbamate [8]. We chose to also use this internationally established BMDL value for the risk assessment presented in this study. Data on consumption of spirits in Mexico for the population older than 15 was obtained from the Global Information System on Alcohol and Health for the year 2004 [25].

## 3. Results

The incidence of ethyl carbamate in different types of spirits from Mexico and Guatemala is detailed in Table 1. There were no statistically significant differences in ethyl carbamate between the different categories in the Mexican samples (p=0.589), nor between clandestinely and commercially produced cuxa from Guatemala (p=0.775). The estimated exposure for agave spirit drinkers in Mexico and cuxa drinkers in Guatemala based on the ethyl carbamate concentrations for the entire collection of samples is summarized in Table 2.

The number of tequila samples allowed us to investigate its categories and sub-groups in greater detail. First, tequila sampled in Germany did not show significant differences from the tequila purchased on the domestic Mexican market (p=0.214). Second, “100% agave” tequila did not differ from “mixto” tequila (mixed, less than 100% agave) (p=0.580). Finally, there were no differences between “blanco”, “joven u oro”, “reposado” and “añejo” tequila (p=0.192) (for details on the tequila categories, see Refs. [18, 26, 27]). Because of the evident absence of differences in sub-groups of tequila, we did not make a distinction between categories in Table 1.

The incidence for ethyl carbamate contamination was higher in Mexico than in Guatemala. However, only four samples in total (two mezcals, one tequila, one bacanora) were above the Canadian limit of 0.15 mg/L for distilled spirits. None of the samples were above the limit for fruit spirits of 0.4 mg/L. The larger number of Mexican samples along with the sampling in different states allowed for a more detailed exposure estimation for drinkers of this category of spirits. Based on an average per capita consumption of 1 litre of pure alcohol in the form of spirits, with the assumption that only agave spirits are consumed, we provide a whole population exposure scenario in Table 3. Here, the average exposure is 6 ng/kg bw/day with a corresponding MOE of 52,560. Additionally, we provide exposure scenarios for individual drinkers, showing exposures for different numbers of drinks per day and for different ethyl carbamate concentrations (Table 4). The corresponding MOEs for these exposures are shown in Table 5. For mean ethyl carbamate content in the beverages, the MOE ranges between 14,400 for one drink per day and 2,880 for five drinks per day.

The ethyl carbamate concentrations in all Guatemalan beverages were below the Canadian limit of 0.15 mg/L for distilled spirits. In twelve of the samples, no ethyl carbamate was detected. Three samples were below the limit of quantitation, and only one had a quantifiable content of ethyl carbamate detected (0.06 mg/L). The focus of our study on one Mayan town in Guatemala did not allow us to assess the public health risk of ethyl carbamate on a countrywide scale. Of significance, in cases of daily consumption of one drink (125 mL at 18% vol) of the sample with the highest concentration would result in an exposure of 125 ng/kg bw/day and a MOE of 2,400.

## 4. Discussion

### 4.1. Mexico

The results of our sampling of 110 Mexican agave spirits are in agreement with the very limited data available in the literature. This includes two studies that evaluated single samples of tequila exported to Australia and Korea, which contained relatively low concentrations of ethyl carbamate (below 0.01 mg/L) [28, 29].

The incidence and concentration of ethyl carbamate in Mexican agave spirits appear to be of a magnitude similar to that of distilled alcoholic beverages on the European market. The EFSA has described an average content of 0.07 mg/L and 95^th^ percentile of 0.29 mg/L for European spirits [8]. This compares well with our average of 0.05 mg/L and 95^th^ percentile of 0.14 mg/L. Interestingly, some European fruit spirits contain significantly higher values of 0.75 mg/L (average) and 3.18 mg/L (95^th^ percentile) [8]. In contrast to certain fruits and sugarcane [30], and taking into consideration the fact the genus has not yet been systematically studied, agave appears to be a non-cyanogenic plant species [31]. For this reason, only the minor ethyl carbamate formation mechanism, which is dependent on yeast strain and urea as a by-product of arginine catabolism [32, 33], appears to be relevant in agave spirits.

In general, a MOE of 10,000 or higher, if based on a BMDL from an animal study, is considered a low public health concern and subsequently a low priority for risk management action [24]. In the case of the whole population risk assessment, the MOEs were above 10,000 for all scenarios (Table 3). The MOEs were only below this threshold for individuals consuming more than two drinks per day. The proportion of Mexicans in 2002 with more than 20 g pure alcohol (about two drinks) per day based on a triangulation of survey and per capita information [34] was estimated to be 20.5% overall, 36.4% in men and 5.8% in women [35].

The limitations of this assessment include sampling in only a sub-group of Mexican States. We focused our sampling on the major production sites (i.e. Jalisco for tequila and Oaxaca for mezcal). However, we deem it to be unlikely that agave alcohol production in other Mexican states would be significantly different from our sample. A further limitation is that we did not include pulque, beer, wine or other spirits from the Mexican market for ethyl carbamate analysis. Our rationale for the exclusion of pulque is the fact that non-distilled fermented beverages with relatively low alcohol content (e.g. beer, wine) generally contain significantly lower ethyl carbamate concentrations than their distilled spirit counterparts [4, 8]. As such, we find it extremely unlikely that higher ethyl carbamate concentrations occur in pulque than in agave spirits, both of which are essentially manufactured from the same raw material. We did not include the other types of alcoholic beverages, as the literature thus far shows a lack of regional dependence for ethyl carbamate contamination in the standard types of alcoholic beverages (beer, wine, international spirits) (see e.g. [4, 8]).

### 4.2. Guatemala

The risk of ethyl carbamate for drinkers of cuxa in our study region of Nahualá appears to be comparably low. The incidence of ethyl carbamate in cuxa, as well as its concentrations were lower than that described by the EFSA for international spirits, e.g. the average ethyl carbamate content in spirits (excluding fruit spirits) from Europe is 0.07 mg/L [8]. The incidence was also lower than in sugarcane spirits (cachaça) from Brazil [36]. This difference may be due to the complete use of sugarcane juice in cachaça production [37], as opposed to partially refined raw sugar in cuxa production [19]. Cyanide is the major precursor for significant ethyl carbamate formation in fruit spirits [6] and cyanogenic substances in sugarcane have also been discussed as precursors of ethyl carbamate during cachaça production [33, 36]. As such, the refined sugarcane material used in cuxa production, which lacks cyanogenic compounds, is a likely explanation for the lowered incidence of ethyl carbamate in this alcohol.

It should be noted that alcohol consumption is a major problem in this community. Liver cirrhosis was the third leading cause of adult death, and first for men in the year 2007 [19]. With a drinking culture characterized by irregular heavy drinking, especially around town market days, alcohol consumption appears to play a major role in cases of violence and domestic abuse, and is linked with street habitation, which is associated with exposure to violence (i.e. assaults) and severe weather, health problems and street inhabiting drinkers [19]. Cuxa, a popular clandestinely produced alcohol, contributes to these problems. Of note, we also sampled this alcohol with the finding of significantly high levels of acetaldehyde [19], a metabolite of ethanol, demonstrated as carcinogenic to animals, and possibly so for humans (classified as IARC Group 2B) [38]. The authors conclude that ethyl carbamate is not a particular health concern for the drinkers of cuxa in Nahualá, nor are reduction measures for this contaminant required. More pressing problems appear to be volume of alcohol consumption along with the patterns of drinking. The prevalent acetaldehyde contamination in cuxa also appears to be a problem of higher priority than ethyl carbamate contamination [19].

## 5. Conclusions

Our risk assessment is in line with the estimates of the EFSA for Europe [8]. For example, an ethyl carbamate exposure of 60 ng/kg bw/day was estimated for spirit consumers in Europe resulting in a MOE of 5,000 [8]. Comparatively, the ethyl carbamate intake from the consumption of the beverages under study does not differ from that in other countries where the incidence of cirrhosis of the liver is lower, therefore, it seems unlikely that ethyl carbamate is a contributing factor. The ethyl carbamate exposure due to alcoholic beverages in the ng-range was also considerably lower than dosages (2.5–7.6 g/day) known to lead to liver damage, based on instances when ethyl carbamate was used as medicine [16].

For these reasons, the author’s are inclined to reject the hypothesis that ethyl carbamate is a likely cause for the more pronounced incidence of liver diseases in Mexico than in other parts of the world. Ethyl carbamate may also not be a major health factor in alcohols produced using completely or partially refined sugarcane materials.

Future research should focus on studying a larger range of constituents and contaminants to explain liver disease incidence in Mexico. Several likely candidates for this line of research were recently pointed out in our pilot study in Lithuania and Hungary [39]. Acetaldehyde, is a particularly interesting research topic, as we found this compound in relatively high concentrations in our earlier study of Mexican agave spirits [18], and a recent risk assessment has shown that acetaldehyde constitutes a potential public health risk - even without consideration for the metabolically produced acetaldehyde [40].

## Figures and Tables

**Table 1:** Incidence of ethyl carbamate contamination in alcoholic beverages from Mexico and Guatemala.

Country	Category	Numbers of samples in the range [mg/L]					
		< LOD (0.01)	< LOQ (0.04)	0.04–0.1	0.1–0.2	0.2–0.3	0.3–0.4
Mexico	Tequila	9	12	18	7	0	0
Mexico	Mezcal	5	0	27	2	1	0
Mexico	Sotol	5	1	9	1	0	0
Mexico	Bacanora	5	0	7	0	0	1
Guatemala	Clandestine Cuxa	9	1	1	0	0	0
Guatemala	Commercial Cuxa	3	2	0	0	0	0

**Table 2:** Ethyl carbamate concentrations in alcoholic beverages from Mexico and Guatemala.

Country	N	Ethyl carbamate [mg/L] a					
		Mean	Median	90^th^ percentile	95^th^ percentile	99^th^ percentile	Maximum
Mexico, different states (agave spirits)	110	0.05	0.04	0.10	0.14	0.22	0.39
Guatemala, Nahualá (cuxa)	16	0.01	0.00	0.03	0.04	0.06	0.06

^a^ Samples below LOD were calculated as zero

**Table 3:** Whole population risk assessment of ethyl carbamate in Mexico. Assumptions: annual per capita consumption of 1 litre of pure alcohol in the form of agave spirits; alcoholic strength of 40% vol; 60 kg person.

	Scenarios for different ethyl carbamate concentrations				
	Mean	Median	90th percentile	95th percentile	99th percentile
Exposure [ng/kg bw/day]	6	5	11	16	25
Margin of Exposure (MOE)	52,560	65,700	26,280	18,771	11,945

**Table 4:** Exposure to ethyl carbamate from agave spirits in Mexico (calculated for a 60 kg person)

Intake scenarios		Exposure for different ethyl carbamate concentration scenarios in spirits [ng/kg bw/day]				
Drinks per day	Volume of spirit consumed [mL]	Mean	Median	90th percentile	95th percentile	99th percentile
1	25	21	17	42	58	92
2	50	42	33	83	117	183
3	75	63	50	125	175	275
4	100	83	67	167	233	367
5	125	104	83	208	292	458

**Table 5:** Margin of Exposure (MOE) for ethyl carbamate due to agave spirits consumption in Mexico. Calculated with BMDL of 0.3 mg/kg bw/day (MOE = BMDL / Exposure).

Intake scenarios		MOE for different exposure scenarios				
Drinks per day	Volume of spirit consumed [ml]	Mean	Median	90th percentile	95th percentile	99th percentile
1	25	14,400	18,000	7,200	5,143	3,273
2	50	7,200	9,000	3,600	2,571	1,636
3	75	4,800	6,000	2,400	1,714	1,091
4	100	3,600	4,500	1,800	1,286	818
5	125	2,880	3,600	1,440	1,029	655
